# Week one FLT-PET response predicts complete remission to R-CHOP and survival in DLBCL

**DOI:** 10.18632/oncotarget.1990

**Published:** 2014-05-19

**Authors:** Ken Herrmann, Andreas K. Buck, Tibor Schuster, Kathrin Abbrederis, Christina Blümel, Ivan Santi, Martina Rudelius, Hans-Jürgen Wester, Christian Peschel, Markus Schwaiger, Tobias Dechow, Ulrich Keller

**Affiliations:** ^1^ Department of Nuclear Medicine, Technische Universität München, Munich, Germany; ^2^ Department of Nuclear Medicine, Universitätsklinikum Würzburg, Würzburg, Germany; ^3^ Department of Epidemiology, Biostatistics and Occupational Health, McGill University, Montreal, Canada; ^4^ III. Medical Department, Technische Universität München, Munich, Germany; ^5^ Institute of Pathology, Technische Universität München, Munich, Germany; ^6^ Institute of Pathology, Universitätsklinikum Würzburg, Würzburg, Germany; ^7^ Oncology Ravensburg, Ravensburg, Germany

**Keywords:** Lymphoma, DLBCL, Positron emission tomography, [18F]Fluorodeoxythymidine, FLT-PET

## Abstract

Despite improved survival in the Rituximab (R) era, a considerable number of patients with diffuse large B-cell lymphoma (DLBCL) ultimately die from the disease. Functional imaging using [^18^F]fluorodeoxyglucose-PET is suggested for assessment of residual viable tumor very early during treatment but is compromised by non-specific tracer retention in inflammatory lesions. The PET tracer [^18^F]fluorodeoxythymidine (FLT) as surrogate marker of tumor proliferation may overcome this limitation. We present results of a prospective clinical study testing FLT-PET as superior and early predictor of response to chemotherapy and outcome in DLBCL. 54 patients underwent FLT-PET prior to and one week after the start of R-CHOP chemotherapy. Repetitive FLT-PET imaging was readily implemented into the diagnostic work-up. Our data demonstrate that the reduction of FLT standard uptake value_mean_ (SUV_mean_) and SUV_max_ one week after chemotherapy was significantly higher in patients achieving complete response (CR, n=48; non-CR, n=6; p<0.006). Martingale-residual and Cox proportional hazard analyses showed a significant monotonous decrease of mortality risk with increasing change in SUV. Consistent with these results, early FLT-PET response showed relevant discriminative ability in predicting CR. In conclusion, very early FLT-PET in the course of R-CHOP chemotherapy is feasible and enables identification of patients at risk for treatment failure.

## INTRODUCTION

CHOP (cyclophosphamide, doxorubicine, vincristine and prednisone) or CHOP-like chemotherapy in combination with the chimeric monoclonal anti-CD20 antibody rituximab (R) is the standard of care in diffuse large B-cell lymphoma (DLBCL). Despite improved overall response rates and progression free (PFS) as well as overall survival (OS) in the R era, a considerable fraction of patients does not achieve a durable remission after first line treatment and will ultimately die from the disease. Therefore, it remains crucial to identify these patients prior to or early in the course of treatment [1-3].

DLBCL is heterogeneous with respect to biology and clinical course. The international prognostic index (IPI) allows estimation of the individual prognosis based on easily available parameters [4] and has remained useful for risk estimation in the rituximab era [5]. Important insights into the molecular biology of this entity have been gained with the introduction of DNA microarrays, which provide a genome-wide profile of mRNA expression levels in cancer samples. Gene expression profiling (GEP) studies support the view that DLBCL is a heterogeneous diagnostic category, as 3 molecular subtypes, termed germinal center B-cell (GCB) DLBCL, activated B-cell (ABC) DLBCL, and primary mediastinal B-cell lymphoma (PMBL), could be detected, which are often indistinguishable using conventional diagnostic tools. These diagnostic DLBCL categories have significantly different survival rates after standard treatment [6-9].

A different attempt to identify patients with higher risk of treatment failure is to perform functional PET imaging. However, while interim FDG-PET has proven useful to identify patients that have an excellent prognosis after standard treatment, this modality has heretofore failed to accurately identify patients who would benefit from alternative treatment strategies or who should be included into clinical trials due to dismal outcome with R-CHOP-like therapy [10-13]. An earlier identification of patients using an alternative radiotracer that allows response assessment immediately after initiation of chemotherapy may be beneficial. A promising candidate is the thymidine analog 3`-deoxy-3`-[^18^F]fluorothymidine (FLT), a PET tracer derived from the cytostatic drug azidovudine (AZT), that allows in vivo imaging of proliferating tissues and malignant tumors [14]. Recently, published studies demonstrated a significant correlation of tumor cell proliferation and FLT uptake in lymphoma and solid tumors [15-21]. Recent studies have also shown that FLT-PET allows non-invasive assessment of tumor grading, very early response assessment, and possibly survival [15, 19, 22-24] in experimental animal models and patients.

Predictive markers are desirable for guiding risk-adjusted treatment in lymphoma. The aim of this prospective study was to assess the suitability of FLT and in particular the decrement of FLT uptake one week after start of immuno-chemotherapy to predict response and clinical outcome in patients with DLBCL.

## RESULTS

### Response to therapy and survival

**Figure 1:**
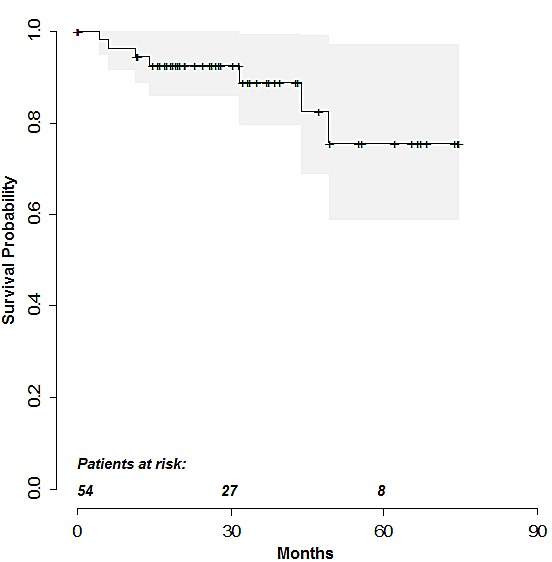
Kaplan-Meier curve depicting estimated overall survival probabilities Shaded area indicates the 95% confidence interval for the survival function. Observation times of individuals without fatal event (censored cases) are marked with the symbol +.

54 patients met the inclusion criteria and completed the full protocol. Staging was performed according to established standards using CT/PET-CT scans as the reference method. Results are shown in Table 1. End of therapy assessment was available in 52 patients and indicated CR in 46 patients, PR in 2 patients, and PD was found in 4 patients, respectively. Importantly, FDG-positivity was found in 5 of the 32 patients with end of treatment FDG-PET, including 4 patients in the non-CR group. Two patients were lost to follow-up. Recurrence and death were reported in 6 (11%) and 7 (13%) patients respectively. The estimated 1-, 3- and 5-year recurrence free survival probabilities (95% confidence interval) were 0.94 (0.89 to >0.99), 0.90 (0.81 to 0.99) and 0.78 (0.59 to >0.99). The respective estimated overall survival probabilities were 0.94 (0.89 to >0.99), 0.89 (0.80 to 0.99) and 0.76 (0.59 to 0.97) (Fig. 1).

**Table 1:** Patient characteristics

Characteristic	Number	Percentage
Age Median Range	62.5 (26-80)	
Histology DLBCL FL grade IIIB	52 2	96 4
IPI score 0/1 2 3 4/5	22 12 12 8	40 23 23 14
Stage I II III IV	14 7 5 28	26 13 10 51

### FLT uptake reduction one week after chemotherapy

Mean uptake of FLT-1 in lymphoma manifestations (mean FLT-1 SUV) was 7.3 (range 1.0 – 18.2). Corresponding maximum FLT-1 uptake values ranged from 1.2 – 20.4, resulting in a mean of FLT-1 SUV_max_ of 9.3. Mean SUV_mean_ decreased one week after start of treatment (FLT-2) to 1.8 (range 0.3 – 7.4) (Table 2), resulting in a mean decrease of 73.1% (range: 0.0% - 95.8%) (Table 2). Corresponding SUV_max_ values of FLT-2 PET were 2.4 (range: 0.4 – 9.6) calculating to a mean decrease of 73.6% (range: 10.8% - 96.1%) for change of SUV_max_ (Table 2,). Thus, FLT PET performed one week after standard treatment results in a substantial decrease in both mean and maximal tracer uptake.

**Table 2:** FLT-1 and FLT-2 parameters FLT-1: represents the FLT parameters before treatment. FLT-2: represents the FLT parameters one week after treatment initiation. ΔSUV: represents the change of SUV measured in FLT-1 to FLT-2 (ΔSUV=100 - [SUV FLT-1 – SUV FLT2] × 100)

Parameter	Mean	Median	Minimum	Maximum
SUVmean FLT-1	7.3	7.0	1.0	18.2
SUVmax FLT-1	9.3	9.0	1.2	20.4
SUVmean FLT-2	1.8	1.2	0.3	7.4
SUVmax FLT-2	2.4	1.5	0.4	9.6
ΔSUVmean	73.1	78.3	0.0	95.8
ΔSUVmax	73.6	77.7	10.8	96.1

### FLT-2 SUV_mean/max_ predict achieving a CR

Next, the FLT-2 data was analyzed for the predictive value of FLT-2 with regard to achieving a CR. Due to the low number of patients with PR or PD, FLT uptake parameters were compared for patients in CR (n=48) and patients in non-CR (n=6). FLT-2 uptake values one week after treatment initiation were significantly lower in patients achieving a CR compared to non-CR patients (mean SUV_mean_: P=0.006; mean SUV_max_: P=0.005, Table 3), the patient group that contained 4 patients with a positive FDG-PET at end of treatment. Thus, FLT uptake one week after treatment is predictive for achieving a CR.

**Figure 2:**
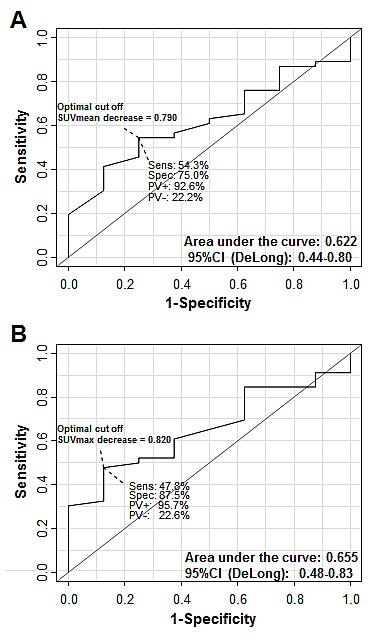
Diagnostic accuracy regarding prediction of complete remission using SUV decrease A, SUVmean decrease. B, SUVmax decrease. Sens: sensitivity; Spec: specificity; PV+: Positive predictive value; PV-: negative predictive value; CI: confidence interval.

We also calculated whether the decrement in either FLT SUV was predictive. The mean decrease of SUV_mean_ and SUV_max_ was higher in patients achieving CR (mean SUV_mean_-decrease: 73.6%, median: 79.7% and mean SUV_max_-decrease: 74.4%, median: 80.1%, Table 3) compared to non-CR patients (mean SUV_mean_-decrease: 65.7%, median 70.5% and mean SUV_max_-decrease: 63.2%, median 65.5%, Table 3). ROC analyses resulted in areas under the curves of 0.622 (95%CI: 0.44-0.80) for SUVmean and 0.655 (95% CI: 0.48 – 0.83) for changes of SUVmax to predict complete response (Fig. 2). Corresponding optimal cut-offs calculated to a decrease of 79.0% SUVmean to predict a CR (positive predictive value: 92.6%) and, respectively, 82.0% SUVmax to predict a CR (positive predictive value: 95.7%).

### Association of FLT uptake parameters and survival

**Figure 3:**
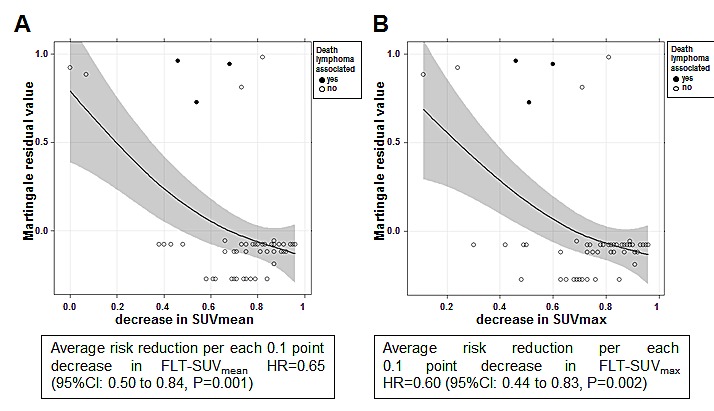
Martingale-residual analysis decrease in A, SUVmean and B, SUVmax and the risk of death in patients achieving a complete remission (CR) or not (PR: partial response; PD: progressive disease). Solid lines depict smoothing functions with 95% confidence bands. Decreasing Martingale-residual values with higher decrease in SUVmean / SUVmax indicate a decline in risk of death with higher SUV decrement.

Six of 54 patients either progressed or relapsed after achieving a response to initial treatment and 7 patients died during the observation period (Fig. 2). Of the 7 deaths 4 were lymphoma-related (7.4%). Martingale-residual analysis was performed for all seven death events and revealed a significant correlation between survival and change of SUV (Fig. 3). The corresponding estimated hazard ratios per one-point increment of FLT-SUV_mean_ and FLT-SUV_max_ were 0.65 (95% CI: 0.50 to 0.84, p=0.001) and 0.60 (95% CI: 0.44 to 0.83, p=0.002) respectively.

**Table 3:** FLT uptake values depending on response

Parameter	Mean	Median	Minimum	Maximum	P-Value
FLT-1 SUVmean non-CR-group	9.3	8.8	6.2	13.7	0.073
FLT-1 SUVmean CR group	7.2	7.0	1.0	18.2	
FLT-1 SUVmax non-CR-group	10.9	10.6	7.0	16.4	0.265
FLT-1 SUVmax CR group	9.2	8.8	1.2	20.4	
FLT-2 SUVmean non-CR-group	3.4	2.2	1.9	7.4	0.006
FLT-2 SUVmean CR group	1.7	1.1	0.3	7.4	
FLT-2 SUVmax non-CR-group	4.2	2.8	2.4	8.9	0.005
FLT-2 SUVmax CR group	2.2	1.4	0.4	9.6	
Decrease SUVmean non-CR-group	65.7	70.5	46.0	78.1	0.089
Decrease SUVmean CR group	73.6	79.7	0.0	95.8	
Decrease SUVmax non-CR-group	63.2	65.5	45.7	78.0	0.064
Decrease SUVmax CR group	74.4	80.1	10.8	96.1	

## DISCUSSION

The thymidine analogue FLT has been shown to reflect proliferation-dependent retention of nucleosides in malignant lymphoma, which can be assessed non-invasively by PET [25]. Several studies testing interim FDG-PET after 2-4 cycles of therapy have heretofore failed to identify patients who would benefit from alternative treatment strategies or who should be included into clinical trials due to dismal outcome with R-CHOP-like therapy [10-13]. Here we used very early interim FLT-PET performed one week after treatment initiation to test the hypothesis that, firstly, the drop in proliferation measured by FLT uptake/retention and, secondly, the remaining FLT uptake (FLT-2 SUV) may allow predicting not only treatment response but also survival.

In concordance with the recent report of Lee and colleagues [24] our similarly designed study (FLT-PET day 7 after cycle 1 vs. before cycle 2) that included a comparable number of patient also points to early FLT-PET assessment as a prognostic/predictive factor with regard to response and survival. Assuming that very early FLT-PET assessment should reflect immediate treatment efficacy we deliberately chose this very short interval between treatment initiation and second FLT-PET imaging. Concluding from the comparison of these two studies however the very early assessment performed at day 7 might be too early and therefore inferior. It is possible that the use of immunotherapy may increase lesion inflammation. Since the mode of supposed action for rituximab involves, next to direct pro-apoptotic or complement-mediated effects [26] also antibody-dependent cellular cytotoxicity via the Fc fragment of the antibody [27], the interval selected in our study could be too short to optimally assess treatment efficacy. Similarly, FDG measurement requires a minimum interval to immuno-chemotherapy due to a transient increase in stromal reaction that may result in overestimation of the fraction of viable cells [28]. The recommendations by the European Organization for Research and Treatment of Cancer consider a time interval of one to two weeks between completion of a chemotherapy cycle and FDG PET optimal to avoid transient flare at the disease sites [29]. Thus a certain delay may be more suitable for FLT-PET, too. On the other hand our study clearly demonstrates in a large cohort that the decrement in FLT uptake briefly after one therapy cycle is quite substantial. We define an optimal cut-off value for SUVmean (79%) and SUVmax (82%) decrease predictive for achieving a complete remission, an important prognostic factor in first line treatment of DLBCL [30].

Besides the above discussed limitations of our study regarding the very early time point of assessment, the moderate power to predict survival is however clearly limited by the bias towards a patient cohort not under immediate treatment pressure. Although synthesis is highly standardized, FLT remains an experimental PET tracer and was therefore only available once a week at our institution. This limitation did not allow us to include more patients with adverse prognostic factors, and reduced performance status due to lymphoma activity is a very important adverse prognostic factor [4]. Including patients selected for adverse prognostic factors determined for example by IPI score (4, 5), molecular assessment (31-33) or also by advanced not yet broadly available techniques (34-37) should allow to define a high risk population more exactly than one single approach alone. Such a high risk patient selection would also come by assessing the suitability of FLT for early prediction of treatment failure in a refractory/relapsed patient cohort [38].

To our knowledge we present results of one of only two prospective studies investigating the predictive value of very early interim FLT-PET regarding response to treatment and survival in aggressive B-NHL. Despite the favourable prognosis and outcome of patients in our series, a significant role of FLT-PET for early survival prediction could be demonstrated. However, although the study results suggest potential usefulness of FLT-PET for response prediction (upper confidence limits of the estimated discrimination indices >0.8), further studies need to be conducted to establish optimal thresholds and to define more precisely the associated predictive performance. Several adjustments with regard to timing and patient selection in upcoming trials investigating FLT or other novel PET tracers will be required.

## METHODS

### Patients

54 patients with biopsy proven aggressive B-cell Non-Hodgkin lymphoma (B-NHL) were included in this prospective study and completed the full FLT-PET imaging protocol (26 men, 28 women, mean age: 58 ± 15 years). All patients were scheduled to undergo systemic chemotherapy with R-CHOP-like treatment. Detailed patient characteristics and risk factors are shown in Table 1.

**Figure 4:**
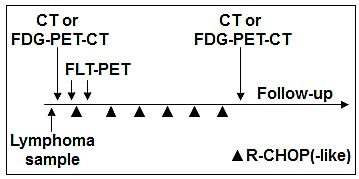
PET imaging and treatment schedule

All 54 patients underwent a pre-therapeutic FLT-PET scan (FLT-1) as well as second scan (FLT-2) one week after start of treatment (Fig. 4). Exclusion criteria included previous or concurrent malignancies, preceding chemo- or radiotherapy, and patients younger than 18 years. Details of the study were explained by a physician and written informed consent was obtained from all patients. The study protocol was approved by the responsible ethics committee of the Technische Universität München.

### Study design

Baseline FLT-PET examination (FLT-1) was performed within 1 week before therapy, together with standard staging modalities (clinical examination, CT). As FDG-PET is not routinely reimbursed in Germany, it was available only for initial staging in a subgroup of patients (n=32). FLT-PET imaging was repeated in all 54 patients around one week after the start of the first course of R-CHOP treatment (FLT-2) (range, day 5 to day 8, mean 6.3 days). An end of therapy FDG-PET as recommended by the revised response criteria published in 2007 was performed in 32 of the patients.

Patients received R-CHOP therapy in standard dose (rituximab 375 mg/m^2^, cyclophosphamide 750 mg/m^2^ day 1, doxorubicin 50 mg/m^2^ day 1, vincristine 1.4 mg/m^2^ day 1 and prednisone 100 mg day 1-5) every 2 or 3 weeks with dose modification or delays according to common standards. 3 patients received additional etoposide 100mg/m^2^ day 1-3 (R-CHOEP) and in one patient liposomal doxorubicin was used with 30 mg/m² per day.

### Histopathological classification

Histology of lymphomas was classified according to the updated WHO classification system [39]. In all 54 patients histopathology revealed aggressive B-NHL (Table 1), including 52 patients with DLBCL and 2 patients with follicular lymphoma (FL) grade 3b.

### PET Imaging

3'-deoxy-3'-[^18^F]fluorothymidine was synthesized as previously described [40]. Imaging was performed on a whole-body high resolution PET scanner (ECAT HR+; Siemens/CTI; Knoxville, TN). This device simultaneously acquires 47 contiguous slices with a slice thickness of 3.4 mm. The in-plane image resolution of transaxial images was approximately 8 mm full width at half maximum (FWHM), with an axial resolution of approximately 5 mm FWHM.

Static emission images were acquired 45 minutes after injection of approximately 300 MBq FLT (range: 270 – 340 MBq). Emission data were corrected for random coincidences, dead time and attenuation and reconstructed by filtered backprojection (Hanning filter with cut-off frequency 0.4 cycle per bin). The matrix size was 128 × 128 pixels with a pixel size of 4.0 × 4.0 mm. The image pixel counts were calibrated to activity concentrations (Becquerel/milliliter) and decay corrected using the time of tracer injection as reference.

### PET Data Analysis

All PET scans were evaluated by two observers (board-certified nuclear medicine specialists) blinded to the clinical data and the results of other imaging studies. Circular regions of interest (ROIs) with a diameter of 1.5 cm were placed in the area with the highest tumor activity, as previously published [41]. Mean lesion diameter and range of initial tumor size were 4.6 cm (median: 4.0, range: 2.0-17.5). Mean standardized uptake values (SUV) were calculated from each ROI using the formula: SUV = measured activity concentration (Bq/g) × body weight (g)/injected activity (Bq). For further analyses, mean values from both observers were used. Side-by-side analysis has been performed to ensure SUV-calculation in identical ROIs at various time points. This algorithm has been demonstrated to be a valuable tool for assessment of treatment response [41, 42].

For definition of ROIs and data analysis, computer programs were developed in the Interactive Data Language (IDL; Research Systems, Inc., Boulder, Co) using the Clinical Application Programming Package (CAPP; Siemens/CTI, Inc.) [43].

### Clinical Evaluation and Follow-up

CT was performed as part of the routine clinical management in all patients. Baseline CT of neck, thorax, abdomen, and pelvis were performed in all patients before chemotherapy. Patients were reevaluated by means of CT after 3 and 6 courses of chemotherapy. The treatment response was classified after completion of 6 cycles of R-CHOP/CHOP as complete response (CR), partial response (PR), no change (NC), or progressive disease (PD) according to the RECIST 1.1 criteria based on the bi-dimensional diameters of corresponding tumor lesions measured by ruler or caliper [44]. Further follow up evaluations were carried out according to standard protocols every 3 months. The median follow-up estimated by the inverse Kaplan-Meier method [45] was 32.2 months (range: 11.5 – 73.2 months; median: 28.6 months). The patient management was not influenced by the results of FLT-PET studies.

### Statistical Analysis

Statistical analyses were performed using PASW Statistics software (version 20.0; SPSS, Inc. Chicago, IL) and the statistical software R [46]. Quantitative values were expressed as mean ± standard deviation or median and range as appropriate. Comparisons of related metric measurements were performed using Wilcoxon-signed rank test and the Mann-Whitney-U test was used to compare quantitative data between two independent samples. To assess prognostic impact of continuous variables with regard to survival, Martingale-residual analysis was performed [47]. Smoothing spline equations have been fitted to the residual plots to depict shape of functional relationship between the continuous prognostic variable and the risk of death.

Fisher's exact test was used for comparison of frequencies and Spearman correlation coefficients were calculated to quantify bivariate correlations of measurement data. Exact two-sided 95 percent confidence intervals (CI) were reported for estimates of sensitivity and specificity. In order to assess the discriminative ability of FLT-SUV decreases for the dichotomous outcome tumor response (complete remission (CR) versus partial response (PR) or progressive disease (PD)), receiver operating characteristics (ROC) curves were fitted and the area under the curve (AUC) along with 95% confidence intervals reported. All statistical tests were conducted two-sided and a p-value less than 0.05 was considered to indicate statistical significance. No correction of p-values was considered in the course of multiple testing; however, results of all performed tests were thoroughly reported, allowing for an informal adjustment for multiplicity while reviewing the data [48].
